# Stress on caregivers providing prolonged mechanical ventilation patient care in different facilities: A cross-sectional study

**DOI:** 10.1371/journal.pone.0268884

**Published:** 2022-05-25

**Authors:** Yeong-Ruey Chu, Chin-Jung Liu, Chia-Chen Chu, Pei-Tseng Kung, Wen-Yu Chou, Wen-Chen Tsai

**Affiliations:** 1 Department of Public Health, China Medical University, Taichung, Taiwan; 2 Department of Respiratory Therapy, China Medical University Hospital, Taichung, Taiwan; 3 School of Nursing, China Medical University, Taichung, Taiwan; 4 Department of Healthcare Administration, Asia University, Taichung, Taiwan; 5 Department of Health Services Administration, China Medical University, Taichung, Taiwan; Stanford University School of Medicine, UNITED STATES

## Abstract

**Purpose:**

Taiwan has implemented an integrated prospective payment program (IPP) for prolonged mechanical ventilation (PMV) patients that consists of four stages of care: intensive care unit (ICU), respiratory care center (RCC), respiratory care ward (RCW), and respiratory home care (RHC). We aimed to investigate the life impact on family caregivers of PMV patients opting for a payment program and compared different care units.

**Method:**

A total of 610 questionnaires were recalled. Statistical analyses were conducted by using the chi-square test and multivariate logistic regression model.

**Results:**

The results indicated no associations between caregivers’ stress levels and opting for a payment program. Participants in the non-IPP group spent less time with friends and family owing to caregiver responsibilities. The results of the family domain show that the RHC group (OR = 2.54) had worsened family relationships compared with the ICU group; however, there was less psychological stress in the RCC (OR = 0.54) and RCW (OR = 0.16) groups than in the ICU group. In the social domain, RHC interviewees experienced reduced friend and family interactivity (OR = 2.18) and community or religious activities (OR = 2.06) than the ICU group. The RCW group felt that leisure and work time had less effect (OR = 0.37 and 0.41) than the ICU group. Furthermore, RCW interviewees (OR = 0.43) were less influenced by the reduced family income than the ICU group in the economic domain.

**Conclusions:**

RHC family caregivers had the highest level of stress, whereas family caregivers in the RCW group had the lowest level of stress.

## Introduction

Prolonged mechanical ventilation (PMV) is defined as the operation of a mechanical ventilation support system for more than 6 hours per day and exceeding 21 days [1, 2]. The PMV incidence rates per 100 ICU admissions in Europe and the US were found to be 5%-11% [3–6]. Taiwan had a PMV rate per 100 mechanical ventilation patients of 20% [7], while China had a PMV rate of 36% [8], and some PMV patients were found to occupy intensive care unit (ICU) beds. To increase the emergency department turnover rate, control medical expenses, and increase the weaning rate of mechanical ventilation, weaning centers (also called subacute or post-acute care) have been established to rapidly liberate patients from the need for mechanical ventilators in the United States [9, 10], Europe [4, 11], and Taiwan [12–14].

The Taiwan National Health Insurance provides a comprehensive payment system for integrated prospective payment plans for PMV patients called integrated prospective payment program (IPP) who are at least 17 years old. This system has integrated payments with managed care systems since July 2000 [2]. This IPP plan covers four types of care and different payments, including ICU for critical care, respiratory care centers (RCCs) for step-down subacute units, respiratory care ward (RCW) for long-term care facilities, and respiratory home care (RHC) for PMV patients [2]. The regulation of IPP are fee-for-service ICU care (within 21 d), per-diem RCC (for up to 42 d), per capitation RCW, and per-month home ventilation service. The ICU, RCC, and RCW belong to the institutions, and 24 hours medical staff are available. The patients who use ventilators at home have nurses and respiratory therapists visit twice a month and physician visit once two months.

The intervention of the IPP has increased the turnover rate of ICU beds and reduced the length of hospital stay. However, PMV patients in RCC, RCW, and RHC have seen a significant increase in the length of required care [13–15]. Studies that as general home care patients are more familiar with the environment at home, they feel more comfortable, amenable, and experience a better than in-house care group [16]. However, it was also found that most PMV patients were elderly patients [17, 18] with poor life function independence [19] and had a greater care burden which directly affected their family caregivers [20–24].

Caregivers’ fear of caring for PMV patients can cause stress on the physical health, mind, and soul of both the patients and caregivers [25]. Moreover, it can impose financial burdens on families. Studies show low willingness in the main caregivers of PMV patients because of the high pressure that the job puts on them [16, 20, 26]. However, there is a shortage of studies exploring whether the burden of main family caregivers with patients at different unit has been affected by the IPP two decades ago. This study aimed to compare the associations between caregivers’ stress levels and opting for a payment program, and compared with the impaction of life on caregivers of PMV patients in the different units.

## Materials and methods

### Questionnaire development

We invited 10 PMV subject care experts from each type of institution in northern, central, and southern Taiwan to constitute a panel of experts. The primary family members of the three PMV patients and three nursing staff members from the same RCW formed a focus group to identify the structure and content of the questionnaire items. The content of the questionnaire included (1) the characteristics of respondents, (2) the characteristics of PMV patients, (3) the effect of care for PMV patients on their families, society, and family finances (S1 File); and (4) relevant information on mechanical ventilation.

The questionnaires have three domains (family, social, and economic). They measured aspects related to their lives by a Likert scale (from strongly agree to strongly disagree) to compare the impact of the IPP and different units of primary caregivers of PMV patients on the stress of life. Then we invited five experts to assess the validity of the questionnaire content following the completion of its design. The average content validity index (CVI) was 0.96 (0.73 to 1.00). The reliability of the questionnaire was measured using the Kuder-Richardson Formula 20 (KR-20). After the questionnaire was tested, the KR-20 coefficient of this study was found to be 0.78.

### Power of sample size

This was a cross-sectional observational study. According to Mohamad Adam Bujang et al.’s recommendation for observational large-sample studies using logistic regression, the minimum sample size needs to be at least 500 [27]. A sufficient total of 601 valid samples were collected in this study.

### Participants

The participants included in this study were at least 20 years of age and served as the main family caregivers of PMV patients from northern, central, and southern Taiwan. According to our definition of the stages of PMV patients, the acute ICU stage was the time in ICU and/or less than 7 days in RCC after transfer from ICU; the subacute stage was ≥30 days of stay in RCC; the chronic stage was ≥30 days of RCW stay; and finally, the RHC was ≥30 days of using a mechanical ventilator at home. The participants were divided into two groups (IPP and none-IPP group) according to whether they had joined IPP.

### Participant consent

Because patients are mostly unconscious, interviewers are required to assist in collecting patients’ clinical information, so the research required medical staff, case managers, or respiratory therapists to recruit those who understood the situation of the subjects to agree to do the interview and explain the parameters to the respondents according to the interview instructions (S2 File). This study was approved by the institutional review board of the China Medical University Hospital (IRB No. CMUH102-REC3-105).

### Statistical analysis

Chi-square tests were used to compare the characteristics of patients with PMV in terms of subject characteristics, mechanical ventilation status, and the difference between the impact on the main caregivers of patients at different stages. Finally, we combined “strongly agree,” “agree” and “no objection” into one group and “disagree” and “strongly disagree” into another group, and used the logistic regression model to compare the differences in stress levels experienced by the main family caregivers for ICU, RCC, RCW, and RHC patients. Statistical significance was set at *p* <0.05, and SAS (version 9.4, SAS Institute Inc., Cary, NC, USA) was used for the analysis.

## Results

### Respondent and patient characteristics

As comprehensive analyses datasets were generated by a previous study [28], including 687 eligible respondents from 64 institutions (6 medical centers, 11 regional hospitals, 23 district hospitals, 15 nursing homes, and 9 home care centers) (S3 File). The study was conducted from November 1, 2013, to April 15, 2014. A total of 601 questionnaires were completed (effective questionnaire response rate 87%) and 50.75% were women, with an average age of 51.88 years. A majority of the responses were provided by children of PMV parents (54.74%). The monthly expense paid by the family for the IPP group was significantly lower than that for the non-IPP group (USD 745 vs. USD 912 respectively) (Table 1). The average age of the PMV patients was 70.76 years. Only 38.77% of patients were in a state of alert consciousness, with 34.78% with near-terminal illnesses. A total of 89.35% of the patients had daily mechanical ventilation of 19–24 hours (Table 2).

**Table 1:** Demographic characteristics of respondents with and without IPP.

Variables	Total N = 601		Non-IPP: N = 207		IPP: N = 394		p value
	N	%	N	%	N	%	
Gender							0.674
Male	296	49.25	99	47.83	197	50.00	
Female	305	50.75	108	52.17	197	50.00	
Age in years							
Mean age (SD)	51.88	(11.84)	53.30	(11.59)	51.13	(11.92)	0.032^#^
Educational level							0.504
None	13	2.16	7	3.38	6	1.52	
≦Grade 9	161	26.79	53	25.60	108	27.41	
High school to college	403	67.05	139	67.15	264	67.01	
Graduate school	24	3.99	8	3.86	16	4.06	
Married							0.636
Yes	480	80.00	170	82.13	310	78.88	
Never	90	15.00	28	13.53	62	15.78	
Been married	30	5.00	9	4.35	21	5.34	
Monthly salary in USD							0.194
<1000	154	25.71	45	21.84	109	27.74	
1000~2000	235	39.23	94	45.63	141	35.88	
2000~3000	125	20.87	42	20.39	83	21.12	
3000~4000	51	8.51	15	7.28	36	9.16	
≥4000	34	5.68	10	4.85	24	6.11	
Religion							0.746
Yes	502	83.53	171	82.61	331	84.01	
No	99	16.47	36	17.39	63	15.99	
Relationship with PMV patient							0.423
Parents	41	6.82	8	3.86	33	8.38	
Couple	111	18.47	42	20.29	69	17.51	
Children	329	54.74	115	55.56	214	54.31	
Children-in-law	59	9.82	21	10.14	38	9.64	
Brothers and sisters	27	4.49	10	4.83	17	4.31	
Grandchildren	17	2.83	7	3.38	10	2.54	
Other	17	2.83	4	1.93	13	3.30	
Can someone take turns taking care of the patient with you?							0.809
No	194	32.28	65	31.40	129	32.74	
Yes	407	67.72	142	68.60	265	67.26	
Average monthly expense (USD) Mean (SD)	801.69	(902.27)	912.61	(601.97)	745.3553	(1017.66)	0.014

IPP: integrated prospective payment program.

One US dollar (USD) was 30 New Taiwan dollars (NTD) on Sep. 2013.

# t-test.

**Table 2:** Demographic characteristics of PMV patients with and without IPP.

Variables	Total N = 601		Non-IPP: N = 207		IPP: N = 394		P value
	N	%	N	%	N	%	
Gender							1.000
Male	304	50.58	105	50.72	199	50.51	
Female	297	49.42	102	49.28	195	49.49	
Age in years							
Mean age (SD)	70.76	(17.23)	73.15	(15.99)	69.51	(17.73)	0.014^#^
Conscious status							0.263
Coma	194	32.28	66	31.88	128	32.49	
Unconsciousness	174	28.95	68	32.85	106	26.90	
Alert	233	38.77	73	35.27	160	40.61	
Cause of respiratory failure							**0.003**
Chronic lung disease	131	21.80	51	24.64	80	20.30	
Central neuropathy	185	30.78	60	28.99	125	31.73	
Catastrophic illnesses	209	34.78	83	40.10	126	31.98	
Other	76	12.65	13	6.28	63	15.99	
Unit							**<0.001**
ICU	150	24.96	60	28.99	90	22.84	
RCC	150	24.96	60	28.99	90	22.84	
RCW	150	24.96	60	28.99	90	22.84	
RHC	151	25.12	27	13.04	124	31.47	
Daily bed-time (hrs.)							**0.004** ^$^
0–6	1	0.17	1	0.48	0	0.00	
7–12	37	6.16	5	2.42	32	8.12	
13–18	26	4.33	6	2.90	20	5.08	
19–24	537	89.35	195	94.20	342	86.80	
Joint IPP and waive copayment (yes)	494	82.20	143	69.08	351	89.09	**<0.001**

# t-test

$ Fisher’s exact test; PMV: prolonged mechanical ventilation; IPP: integrated prospective payment program; ICU: intensive care unit; RCC: respiratory care center; RCW: respiratory care ward; RHC; respiratory home care.

### Impact of caring for PMV patients on families

Significantly more caregivers in the non-IPP group (*p* = 0.039), compared with the IPP group, reported reduced time with friends and family. In addition, there were no significant statistical differences in other factors (Table 3).

**Table 3:** Comparison of the impact of IPP for PMV patients on the life of caregivers.

Variables	All		Non-IPP		IPP		*p* value
	N	%	N	%	N	%	
**Total**	601	100	207	34.44	394	65.56	
**[Family domain]**							
**Taking care of the patient worsens the relationship between family members.**							**0.199**
strongly disagree	112	18.64	37	17.87	75	19.04	
disagree	227	37.77	81	39.13	146	37.06	
no objection	91	15.14	37	17.87	54	13.71	
agree	133	22.13	36	17.39	97	24.62	
strongly agree	38	6.32	16	7.73	22	5.58	
**I feel that family life is affected because of caring for the patient**							**0.195**
strongly disagree	47	7.82	14	6.76	33	8.38	
disagree	121	20.13	53	25.60	68	17.26	
no objection	76	12.65	25	12.08	51	12.94	
agree	285	47.42	91	43.96	194	49.24	
strongly agree	72	11.98	24	11.59	48	12.18	
**I experience physical stress because of caring for the patient**							**0.051**
strongly disagree	46	7.65	12	5.80	34	8.63	
disagree	132	21.96	59	28.50	73	18.53	
no objection	98	16.31	28	13.53	70	17.77	
agree	242	40.27	79	38.16	163	41.37	
strongly agree	83	13.81	29	14.01	54	13.71	
**I feel psychologically stressed from caring for the patient**							**0.651**
strongly disagree	27	4.49	9	4.35	18	4.57	
disagree	83	13.81	32	15.46	51	12.94	
no objection	56	9.32	22	10.63	34	8.63	
agree	306	50.92	97	46.86	209	53.05	
strongly agree	129	21.46	47	22.71	82	20.81	
**[Social domain]**							
**I feel that my time for my friends and family has reduced because of caring for the patient**							**0.039**
strongly disagree	36	5.99	12	5.80	24	6.09	
disagree	126	20.97	56	27.05	70	17.77	
no objection	121	20.13	31	14.98	90	22.84	
agree	247	41.10	82	39.61	165	41.88	
strongly agree	71	11.81	26	12.56	45	11.42	
**I feel that the time for community or religious activities has reduced because of caring for the patient**							**0.170**
strongly disagree	37	6.16	13	6.28	24	6.09	
disagree	124	20.63	53	25.60	71	18.02	
no objection	144	23.96	50	24.15	94	23.86	
agree	225	37.44	66	31.88	159	40.36	
strongly agree	71	11.81	25	12.08	46	11.68	
**I feel that leisure time has decreased because of caring for the patient**							**0.125**
strongly disagree	28	4.66	9	4.35	19	4.82	
disagree	88	14.64	39	18.84	49	12.44	
no objection	95	15.81	38	18.36	57	14.47	
agree	286	47.59	88	42.51	198	50.25	
strongly agree	104	17.30	33	15.94	71	18.02	
**My work is affected because of caring for the patient**							**0.392**
strongly disagree	34	5.66	13	6.28	21	5.33	
disagree	113	18.8	47	22.71	66	16.75	
no objection	118	19.63	41	19.81	77	19.54	
agree	213	35.44	66	31.88	147	37.31	
strongly agree	123	20.47	40	19.32	83	21.07	
**It is difficult to find proper social support or assistance to take care of the patient**							**0.085**
strongly disagree	37	6.16	9	4.35	28	7.11	
disagree	149	24.79	63	30.43	86	21.83	
no objection	132	21.96	40	19.32	92	23.35	
agree	206	34.28	65	31.40	141	35.79	
strongly agree	77	12.81	30	14.49	47	11.93	
**[Economic domain]**							
**Reduced family income due to inability to work owing to caregiving responsibilities**							**0.344**
strongly disagree	42	6.99	11	5.31	31	7.87	
disagree	110	18.30	46	22.22	64	16.24	
no objection	113	18.80	40	19.32	73	18.53	
agree	216	35.94	72	34.78	144	36.55	
strongly agree	120	19.97	38	18.36	82	20.81	
**I am under financial pressure because of the cost of caring for the patient**							**0.446**
strongly disagree	21	3.49	7	3.38	14	3.55	
disagree	62	10.32	28	13.53	34	8.63	
no objection	105	17.47	33	15.94	72	18.27	
agree	260	43.26	88	42.51	172	43.65	
strongly agree	153	25.46	51	24.64	102	25.89	

IPP: integrated prospective payment program; PMV: prolonged mechanical ventilation.

### Impact of socioeconomic conditions, caregiver occupation, underlying diseases of the patients on caregiver stress levels and family relationships

We used family income and education level to represent the socioeconomic conditions of caregivers. We then compared the family income and education level of the caregivers of PMV patients at four stages with the psychological pressure and family relationship using Spearman’s rank correlation. In addition, we used the Chi-square test to analyze the patient’s underlying disease, psychological pressure on the caregiver, and family relationships. The results showed that there was no significant difference in the family income of caregivers between psychological stress and family relationships. However, we found that the higher the education level, the lower the adverse effect on the relationship between family members; moreover, this difference was statistically significant (p<0.05) (see Table 4). There was no significant correlation between the underlying disease of PMV patients and the psychological stress on caregivers and family relationships (Table 5).

**Table 4:** The socioeconomic conditions and occupation of caregiver’s impact on stress level and family relationship.

Variables	Family income of caregiver		Education of caregiver	
	correlation coefficient	p-value^1^	correlation coefficient	p-value^1^
Family relationship	-0.017	0.677	-0.166	<0.001
Psychologically stressed	0.008	0.850	-0.080	0.050

^1^Spearman’s rank correlation test.

**Table 5:** Association of underlying diseases of the patients with stress level and family relationship.

Underline disease	Chronic pulmonary disease		Central neuropathy		Catastrophic illness		Others		P-value^1^	
	n	%	n	%	N	%	n	%		
**Psychologically stressed**										**0.515**
strongly disagree	4	3.05	9	4.86	9	4.31	5	6.58		
disagree	17	12.98	22	11.89	37	17.70	7	9.21		
no objection	12	9.16	12	6.49	22	10.53	10	13.16		
agree	67	51.15	99	53.51	104	49.76	36	47.37		
strongly agree	31	23.66	43	23.24	37	17.70	18	23.68		
Total	131	21.80	185	30.78	209	34.78	76	12.65		
**Family relationship**										**0.129**
strongly disagree	19	14.50	38	20.54	38	18.18	17	22.37		
disagree	51	38.93	64	34.59	89	42.58	23	30.26		
no objection	19	14.50	22	11.89	39	18.66	11	14.47		
agree	32	24.43	50	27.03	34	16.27	17	22.37		
strongly agree	10	7.63	11	5.95	9	4.31	8	10.53		
Total	131	21.80	185	30.78	209	34.78	76	12.65		

^**1**^ **Chi-square test.**

**Fig 1:**
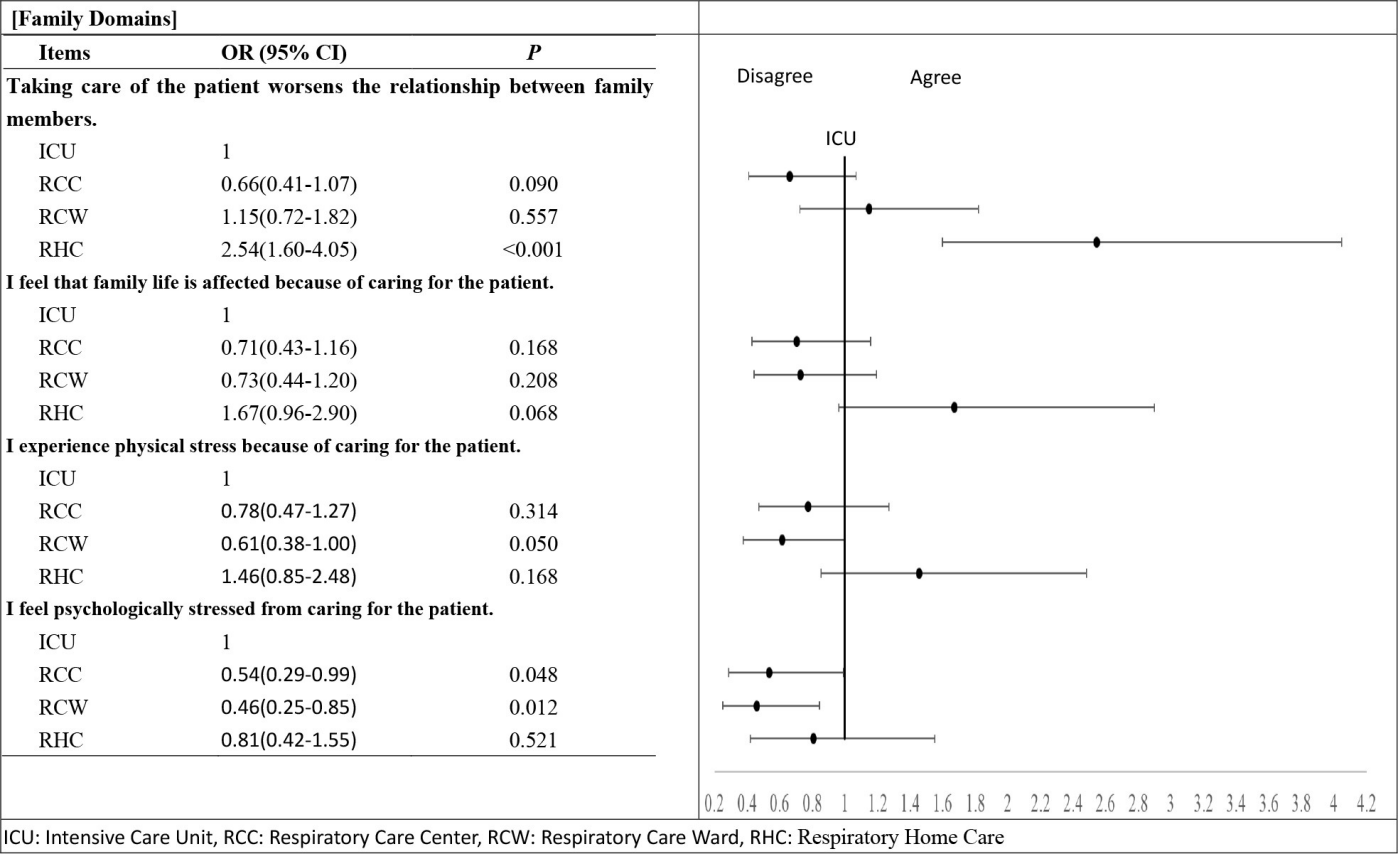
Odds of agreement in the life impact of the caregivers of PMV patients at various stages at family domain.

Regarding the impact on the family caregivers’ life among the ICU, RCC, RCW and RHC groups, results showed that respondents from the RHC group showed more agreement to the item, “Taking care of the patient worsens the relationship between family members” than interviewees in the ICU group (OR = 2.54 (95%CI 1.60–4.05), *p*<0.001); however, respondents from the RCW group showed less agreement to the item, “I experienced physical stress because caring for the patient” than those in the ICU group (OR = 0.61 (95%CI 0.38–1.00), *p* = 0.05). For the item, “I feel psychologically stressed from caring for the patient,” respondents from the RCC (OR = 0.54 (95%CI 0.29–0.99), *p* = 0.048) and RCW (OR = 0.46 (95%CI 0.25–0.85), *p* = 0.012) groups showed less agreement than those from the ICU group (Fig 1) (S4 File).

**Fig 2:**
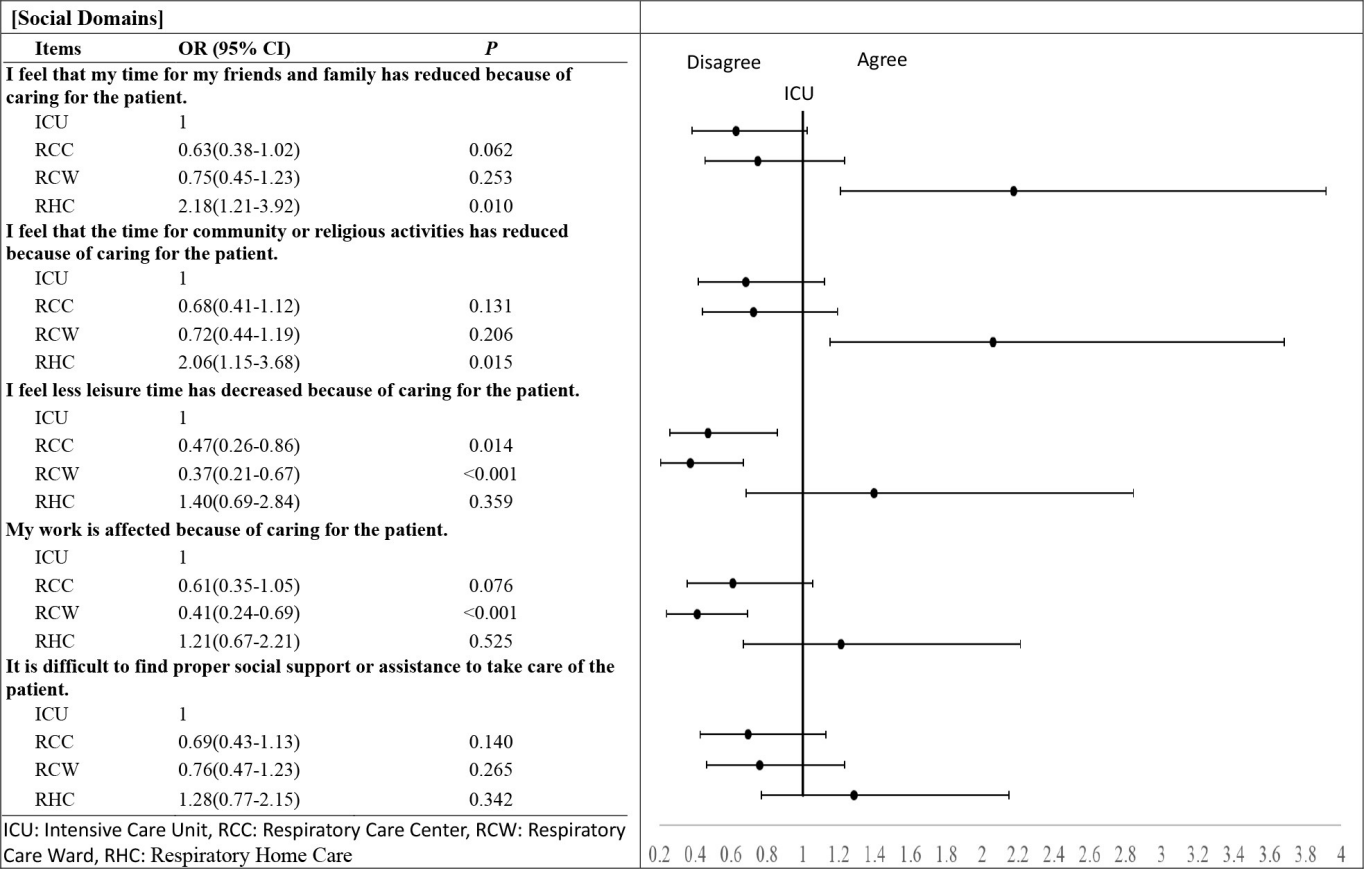
Odds of agreement in the life impact of the caregivers of PMV patients at various stages at social domain.

In the social domain, for items such as “I feel that my time for my friends and family has reduced because of caring for the patient” and “I feel that the time for community or religious activities has reduced because of caring for the patient,” caregivers in the RHC group showed more agreement than those in the ICU group (OR = 2.18 (95%CI 1.21–3.92), *p* = 0.01 and OR = 2.06 (95%CI 1.15–3.68), *p* = 0.014, respectively). For items such as “I feel that leisure time has decreased because of caring for the patient,” the RCC and RCW groups were in less agreement than those in the ICU group (RCC, OR = 0.47 (95%CI 0.26–0.86), *p* = 0.014 and RCW, OR = 0.37 (95%CI 0.21–0.67, *p*<0.001). For the item, “My work is affected because of caring for the patient.” the RCW group was in less agreement than the ICU group (OR = 0.41 (95%CI 0.24–0.69), *p*<0.001) (Fig 2) (S4 File).

**Fig 3:**
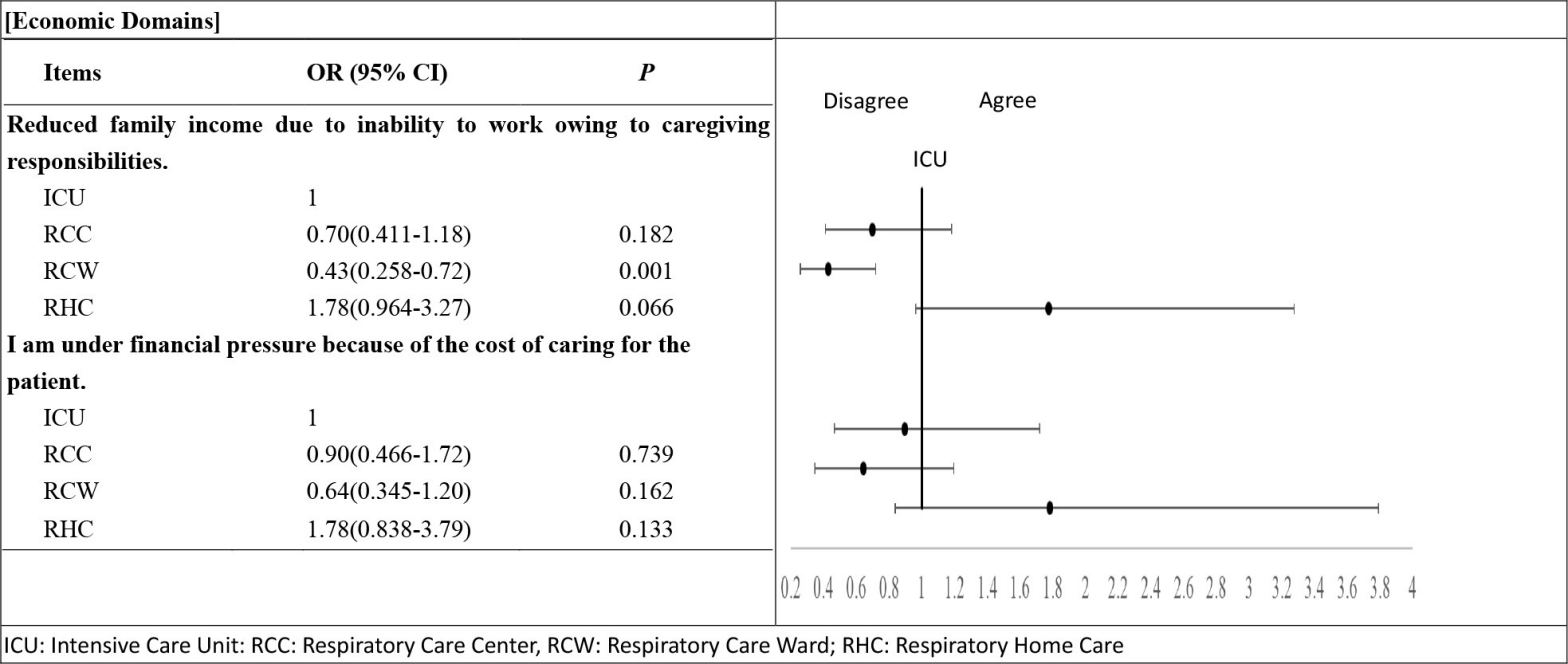
Odds of agreement in the life impact of the caregivers of PMV patients at various stages at economic domain.

In the economic domain, the ICU group (OR = 0.43 (95%CI 0.26–0.72), *p* = 0.001) (Fig 3) (S4 File) had a greater level of agreement with the item “Reduced family income due to inability to work owing to caregiving responsibilities,” than the RCW group.

## Discussion

The PMV patients and respondents had an average age of 70.76 and 51.88 years respectively. Of the main caregivers, 54.74% were children, and 80% of them were married. In the IPP group, the monthly expenses spent on patients were significantly lower than those of the non-IPP group. The caregivers of home-based patients experienced higher stress levels than caregivers of patients in another stage. The caregivers of ICU patients also experienced high stress levels, but only second to those taking care of home-based patients. Furthermore, it was found that caregivers of RCW patients had significantly lower stress levels than the family caregivers of patients in other situations.

This study demonstrated that the stress levels experienced by caregivers of home-based PMV patients (RHC) were 1.21–2.54 times that of their counterparts who care for ICU patients. According to the results, caregivers of home-based PMV patients had poorer sleep quality [29], higher risks of depression, and poorer health conditions [30, 31]. We also found that although caregivers of home-based patients had higher stress levels than those of RCW patients [29], the difference was not significant. As demonstrated in our study, the stress level of the caregivers of home-based patients was the highest, followed by that of caregivers of ICU, RCC, and RCW patients. Therefore, apart from continued attention paid to the stress of the main caregivers of home-based PMV patients [20, 22, 29, 30, 32, 33], we cannot ignore the stress experienced by caregivers of ICU patients.

The results showed that caregivers of ICU and RHC patients had higher stress levels than those of RCC and RCW patients. This might be caused by uncertainties in emergency medical treatments performed to save the lives of ICU patients [34–37] and the pressure to make life-making decisions on behalf of the patients [38]. These caregivers may have a higher risk of developing depression [39] because their stress levels increase with the severity of the patients’ condition [40]. Although RCC and RCW patients were mechanical ventilator users, they had already experienced the uncertainty of the acute phase. At this point, their family members could support each other in caring for patients using only shared knowledge for methods of care. As the caregivers no longer needed to attend to patients 24 hours a day, the stress they experienced was significantly lower than that experienced by caregivers of home-based patients [29].

Since the main caregivers of home-based PMV patients needed to take care of the patients, they had to give up part of their time with family, their comforts of life, and their opportunities to communicate and build relationships with friends and relatives [41]. Notwithstanding, patients with PMV have a poor prognosis [42]. The considerable expense and physical fatigue can aggravate psychological and physical stress experienced by family members, as well as increase socioeconomic burdens over time [21–23, 31, 34]. As a result, most caregivers of PMV patients choose to work with medical institutions instead of taking total care of patients themselves [29, 43]. The caregivers of RCW patients had significantly lower stress levels than those of patients in other situations, indicating that medical institutions were of great help in relieving caregivers’ stress. Therefore, PMV care centers in medical institutions can help family caregivers get plenty of rest and reduce the stress they experience [34].

The strengths of our study conducted a large number of participants from 64 institutions. However, some limitations were considered. First, the impact of the socioeconomic condition of participants on stress level on family is not clear. Second, we used income and education to represent the socioeconomic condition which might not be a complete evaluation.

## Conclusions

Family caregivers of RHC patients had the highest level of stress, followed by their counterparts who care for ICU patients; further, the stress levels of caregivers are not associated with IPP. Higher the education level, the lower the adverse effect on the relationship between family members. Future studies to investigate the relationship between socioeconomic condition and stress level is suggested.

## Supporting information

S1 File Question about the impact of taking care of prolonged mechanical ventilation patients on life (English and Chinese version). (DOCX)

S2 File Interviewees manual (English and Chinese version). (DOCX)

S3 File 64 Interviewee’s institutions information. (DOCX)

S4 File The impact on the family caregivers’ life between the ICU, RCC, RCW and RHC groups. (DOCX)

## Abbreviations

PMV: Prolonged Mechanical Ventilation
IPP: Integrated Payment program
ICU: intensive care unit
RCC: respiratory care centers
RCW: respiratory care ward
RHC: Respiratory home care
