# Supplementary material for: Stress on caregivers providing prolonged mechanical ventilation patient care in different facilities: A cross-sectional study
Source: PLoS One. 2022 May 25;17(5):e0268884. doi: 10.1371/journal.pone.0268884 (PMC9132287; doi:10.1371/journal.pone.0268884)
Supplement: S1 File — (DOCX) [file pone.0268884.s001.docx]

**Comparison of the Impact of IPP for PMV Patients on the Life of Family Caregivers**

Please compare the following situations before and after taking care of the patient, and choose an answer that best suits your situation. ("Strongly agree" means the greater the degree of impact; "Strongly disagree" means not affected)

| Question item | Strongly agree | Agree | no objection | Disagree | Strongly disagree |
| --- | --- | --- | --- | --- | --- |
| **【Family Domain】** |  |  |  |  |  |
| 1. **Taking care of the patient worsens the relationship between family members.** | □ | □ | □ | □ | □ |
| 1. **I feel that family life is affected because of caring for the patient.** | □ | □ | □ | □ | □ |
| 1. **I experience physical stress because of caring for the patient.** | □ | □ | □ | □ | □ |
| 1. **I feel psychologically stressed from caring for the patient.** | □ | □ | □ | □ | □ |
| 【**Social Domain**】 |  |  |  |  |  |
| 1. **I feel that my time for my friends and family has reduced because of caring for the patient.** | □ | □ | □ | □ | □ |
| 1. **I feel that the time for community or religious activities has reduced because of caring for the patient.** | □ | □ | □ | □ | □ |
| 1. **I feel that leisure time has decreased because of caring for the patient.** | □ | □ | □ | □ | □ |
| 1. **My work is affected because of caring for the patient.** | □ | □ | □ | □ | □ |
| 1. **It is difficult to find proper social support or assistance to take care of the patient.** | □ | □ | □ | □ | □ |
| 【**Economic Domain**】 |  |  |  |  |  |
| 1. **Reduced family income due to inability to work owing to caregiving responsibilities.** | □ | □ | □ | □ | □ |
| 1. **I am under financial pressure because of the cost of caring for the patient.** | □ | □ | □ | □ | □ |

IPP: integrated prospective payment program; PMV: prolonged mechanical ventilation

**「健保論質計酬之全民健康保險呼吸器依賴患者整合性照護前瞻性支付方式試辦計畫成效評估計畫」調查問卷**

**第一部份 照顧呼吸器依賴患者對生活之影響**

請您將下列各種情況，和照顧病人之前的情況做比較，請選擇一種最符合您情況的答案。(「非常同意」表示受影響程度越大；「非常不同意」表示不受影響)

| 題 項 | 非常同意 | 同意 | 沒意見 | 不同意 | 非常不同意 |
| --- | --- | --- | --- | --- | --- |
| 【**家庭層面**】 |  |  |  |  |  |
| 因照顧病人而感到家人的關係變得不好 | □ | □ | □ | □ | □ |
| 因照顧病人而感到家庭生活受到影響 | □ | □ | □ | □ | □ |
| 因照顧病人而感到體力不堪負荷 | □ | □ | □ | □ | □ |
| 因照顧病人而感到心理壓力沈重 | □ | □ | □ | □ | □ |
| 【**社會層面**】 |  |  |  |  |  |
| 因照顧病人而感到與親友交往的時間減少 | □ | □ | □ | □ | □ |
| 因照顧病人而感到參與社團或宗教活動減少 | □ | □ | □ | □ | □ |
| 因照顧病人而感到休閒活動減少 | □ | □ | □ | □ | □ |
| 因照顧病人而影響工作 | □ | □ | □ | □ | □ |
| 當照顧病人有困難時，感到不容易得到社會適當的支援或協助(包含親戚、朋友、社團、政府…) | □ | □ | □ | □ | □ |
| 【**經濟層面**】 |  |  |  |  |  |
| 因照顧病人不能外出工作，使家庭收入減少 | □ | □ | □ | □ | □ |
| 因照顧病人的花費很大，讓我感到經濟壓力 | □ | □ | □ | □ | □ |
