# Supplementary material for: Stress on caregivers providing prolonged mechanical ventilation patient care in different facilities: A cross-sectional study
Source: PLoS One. 2022 May 25;17(5):e0268884. doi: 10.1371/journal.pone.0268884 (PMC9132287; doi:10.1371/journal.pone.0268884)

**Interviewee's Manual**

This study was commissioned by the National Health Insurance Administration of the Ministry of Health and Welfare. It is mainly intended to understand whether there is knowledge and acceptance of hospice care and the stress of financial, family, and social differences between the main family caregiver of prolonged mechanical ventilation patients at each stage who have joined the integrated prospective payment program. A questionnaire survey will be conducted in hospitals or institutions that provide respiratory care in the northern, central, and southern parts of Taiwan. The respondents who were over 20 years old mainly took care of family members (excluding caregivers and friends) for patients who received prolonged mechanical ventilation (continuous use of respirators for more than 21 days and over 17 years of age). Questionnaires were collected from at least 600 questionnaires will be collected in Taiwan.

This study was reviewed and approved by the Institutional Review Board on Humanities and Social Science Research of China Medical University and Hospital Research Ethics Committee. The researchers were nurses, case managers, or respiratory therapists who were appointed to confirm patients who met the above conditions. The researchers then invited their family members (respondents) to make appointments. After the interviewer initially agreed, she/he gave the interviewee a complete description of the plan, explained and answered the interviewee’s questions, and then completed the questionnaire by self-write or face-to-face interviews with their consent. This questionnaire is expected to take approximately 5-10 minutes. If the respondent disagrees with the questionnaire survey, this will not affect his or her right to medical treatment in the future. Respondents can withdraw their consent at any time and withdraw from the study without any reason after giving oral consent.

**The research team interviewee's standard work document explaining the agreement**

Hello, I am.. [Organization name + title name + name]. We are entrusted by the National Health Insurance Administration of the Ministry of Health and Welfare, to understand whether there is knowledge and acceptance of hospice care and the stress of financial, family, and social differences between the main family caregiver of prolonged mechanical ventilation patients at each stage who have joined the integrated prospective payment program, and related influencing factors. There is a short questionnaire that takes approximately 5 to 10 minutes. This is an "anonymous" questionnaire. If you feel any discomfort during the answering process, or for any reason, as long as you want to interrupt the questionnaire survey, you can always asking "I want to withdraw from the research". This study is expected to conduct 600 questionnaire surveys across Taiwan. I wonder if you are willing to participate?

**[After the questionnaire survey was completed]**

Thank you for your comments. If you have any questions about the questionnaire survey above, you can call (04)22053366 ext. 6302, the principal investigator, Professor Wen-Chen Tsai, at the Department of Health Services Administration of China Medical University.


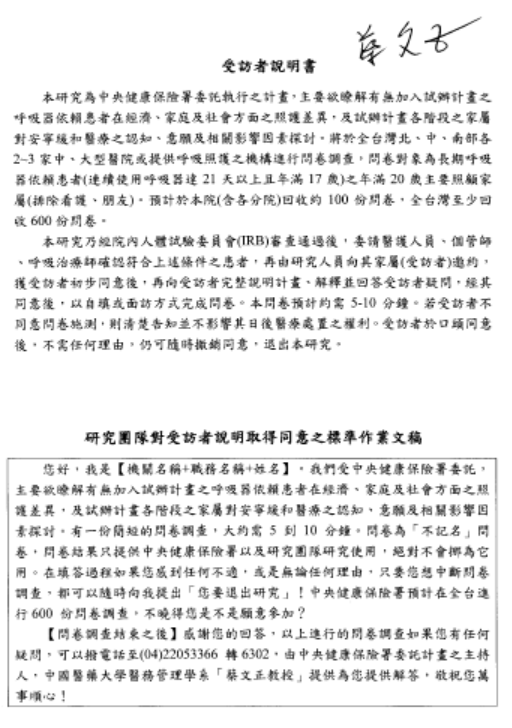

Supplement: S2 File — (DOCX) [file pone.0268884.s002.docx]
