# Supplementary material for: Stress on caregivers providing prolonged mechanical ventilation patient care in different facilities: A cross-sectional study
Source: PLoS One. 2022 May 25;17(5):e0268884. doi: 10.1371/journal.pone.0268884 (PMC9132287; doi:10.1371/journal.pone.0268884)
Supplement: S3 File — (DOCX) [file pone.0268884.s003.docx]

**Supplementary Information for the participating hospitals/institutions**

1. **Linkou Chang-Geng Memorial Hospital**
2. **Ban-Qiao Cathay Hospitat**
3. **Pan-Chiao Cathy hospital attached nursing home**
4. **En-Hua Hospital**
5. **Yi Min Hospital**
6. **WelGong Memorial Hospital**
7. **Kao general hospital**
8. **Taipei Medical University Hospital**
9. **Taipei Veterans General Hospital.**
10. **Gandau Hospital**
11. **Taipei Municipal Wanfang Hospital**
12. **Shuang Ho Hospital, Ministry of Healht and Welfare**
13. **Taipei Hospital, Ministry of Health and Welfare**
14. **Tri-Service General Hospital**
15. **Miaoli Xinsheng Hospital**
16. **Da Chuan Hospital**
17. **Dachien Hospital**
18. **Farun Hospital**
19. **Coundry Hospital**
20. **Tai An Hospital Shuang-Shih Branch**
21. **China Medical University Hospital**
22. **China Medical University Hospital East District Hospital**
23. **Tung's Taichung MetroHarbor Hospital**
24. **Bentang Cheng Ching Hospital**
25. **Bentang Cheng Ching hospital attached nursing home**
26. **ChangAn Hospital attached nursing home**
27. **Show Chwan Memorial Hospital**
28. **Chu Shang Show Chwan Hospital**
29. **China Medical University Beigang Hospital**
30. **Tyz-Yo Hospital attached nursing home**
31. **Keelung Hospital, Ministry of Health and Welfare**
32. **Chiu Hospital**
33. **Tainan Municipal An-Nan Hospital-China Medical University**
34. **Nantou Hospital**
35. **Chia-Yi Christian Hospital attached Ditmanson Medical Foundation Nursing Home**
36. **Chi-Fu Nursing home**
37. **Fuxing nursing home**
38. **De'an Respiration home care**
39. **Meishan Respiration home care**
40. **Jiahe nursing home**
41. **Cikang Nursing Home**
42. **Anfu Home Care Center**
43. **Kangfu Nursing Home**
44. **Kaohsiung Chang Gung Memorial Hospital**
45. **Yuan's General Hospital**
46. **Yuoshen hospital**
47. **Yuoshen Hospital attached nursing home**
48. **Xiao Zhiwen Hospital**
49. **Sheng Gong Hospital**
50. **Hexin Nursing Home**
51. **An Xin Nursing Home**
52. **Yansong Nursing Home**
53. **Quansheng hospital**
54. **Meishan Nursing Home**
55. **Xinsheng Hospital attached nursing home**
56. **Suncheon Hospital**
57. **Suncheon Hospital attached nursing home**
58. **Lian'an Hospital**
59. **Taichung Veterans General Hospital**
60. **Kaohsiung City United Hospital**
61. **Minsheng Hospital**
62. **Xinren Hospital attached nursing home**
63. **Linxin Hospital attached nursing home**
64. **Taipei Public Health Center**
