# Supplementary material for: Stress on caregivers providing prolonged mechanical ventilation patient care in different facilities: A cross-sectional study
Source: PLoS One. 2022 May 25;17(5):e0268884. doi: 10.1371/journal.pone.0268884 (PMC9132287; doi:10.1371/journal.pone.0268884)
Supplement: S4 File — (DOCX) [file pone.0268884.s004.docx]

| **S4: The impact on the family caregivers’ life between the ICU, RCC, RCW and RHC groups** | | | | | | | | | | | | |
| --- | --- | --- | --- | --- | --- | --- | --- | --- | --- | --- | --- | --- |
| **Domain** | **Variables** | **Total** | | **ICU** | | **RCC** | | **RCW** | | **Home** | | **P-value*** |
|  |  | **N** | **%** | **N** | **%** | **N** | **%** | **N** | **%** | **N** | **%** |  |
| **Family 1** | **Taking care of the patient worsens the relationship between family members.** | | | | | | | | | | | |
|  | Disagree | **339** | **56.41** | **91** | **60.67** | **105** | **70.00** | **86** | **57.33** | **57** | **37.75** | **<0.001** |
|  | Agree | **262** | **43.59** | **59** | **39.33** | **45** | **30.00** | **64** | **42.67** | **94** | **62.25** |  |
| **Family 2** | **I feel that family life is affected because of caring for the patient.** | | | | | | | | | | | |
|  | Disagree | **168** | **27.95** | **40** | **26.67** | **51** | **34.00** | **50** | **33.33** | **27** | **17.88** | **0.006** |
|  | Agree | **433** | **72.05** | **110** | **73.33** | **99** | **66.00** | **100** | **66.67** | **124** | **82.12** |  |
| **Family 3** | **I experience physical stress because of caring for the patient.** | | | | | | | | | | | |
|  | Disagree | **178** | **29.62** | **41** | **27.33** | **49** | **32.67** | **57** | **38.00** | **31** | **20.53** | **0.007** |
|  | Agree | **423** | **70.38** | **109** | **72.67** | **101** | **67.33** | **93** | **62.00** | **120** | **79.47** |  |
| **Family 4** | **I feel psychologically stressed from caring for the patient.** | | | | | | | | | | | |
|  | Disagree | **110** | **18.30** | **19** | **12.67** | **32** | **21.33** | **36** | **24.00** | **23** | **15.23** | **0.040** |
|  | Agree | **491** | **81.70** | **131** | **87.33** | **118** | **78.67** | **114** | **76.00** | **128** | **84.77** |  |
| **Social 1** | **I feel that my time for my friends and family has been reduced because of caring for the patient.** | | | | | | | | | | | |
|  | Disagree | **162** | **26.96** | **39** | **26.00** | **54** | **36.00** | **48** | **32.00** | **21** | **13.91** | **<0.001** |
|  | Agree | **439** | **73.04** | **111** | **74.00** | **96** | **64.00** | **102** | **68.00** | **130** | **86.09** |  |
| **Social 2** | **I feel that the time for community or religious activities has been reduced because of caring for the patient.** | | | | | | | | | | | |
|  | Disagree | **161** | **26.79** | **39** | **26.00** | **51** | **34.00** | **49** | **32.67** | **22** | **14.57** | **<0.001** |
|  | Agree | **440** | **73.21** | **111** | **74.00** | **99** | **66.00** | **101** | **67.33** | **129** | **85.43** |  |
| **Social 3** | **I feel that leisure time has decreased because of caring for the patient.** | | | | | | | | | | | |
|  | Disagree | **116** | **19.30** | **20** | **13.33** | **37** | **24.67** | **44** | **29.33** | **15** | **9.93** | **<0.001** |
|  | Agree | **485** | **80.70** | **130** | **86.67** | **113** | **75.33** | **106** | **70.67** | **136** | **90.07** |  |
| **Social 4** | **My work is affected because of caring for the patient.** | | | | | | | | | | | |
|  | Disagree | **147** | **24.46** | **28** | **18.67** | **41** | **27.33** | **54** | **36.00** | **24** | **15.89** | **<0.001** |
|  | Agree | **454** | **75.54** | **122** | **81.33** | **109** | **72.67** | **96** | **64.00** | **127** | **84.11** |  |
| **Social 5** | **It is difficult to find proper social support or assistance to take care of the patient.** | | | | | | | | | | | |
|  | Disagree | **186** | **30.95** | **43** | **28.67** | **55** | **36.67** | **52** | **34.67** | **36** | **23.84** | **0.066** |
|  | Agree | **415** | **69.05** | **1074** | **71.33** | **95** | **63.33** | **98** | **65.33** | **115** | **76.16** |  |
| **Economic 1** | **Reduced family income due to inability to work owing to caregiving responsibilities.** | | | | | | | | | | | |
|  | Disagree | **152** | **25.29** | **32** | **21.33** | **42** | **28.00** | **58** | **38.67** | **20** | **13.25** | **<0.001** |
|  | Agree | **449** | **74.71** | **118** | **78.67** | **108** | **72.00** | **92** | **61.33** | **131** | **86.75** |  |
| **Economic 2** | **I am under financial pressure because of the cost of caring for the patient.** | | | | | | | | | | | |
|  | Disagree | **83** | **13.81** | **20** | **13.33** | **22** | **14.67** | **29** | **19.33** | **12** | **7.95** | **0.040** |
|  | Agree | **518** | **86.19** | **130** | **86.67** | **128** | **85.33** | **121** | **80.67** | **139** | **92.05** |  |
| **Abbreviations: ICU, intensive care unit; RCC, Respiratory Care Center; RCW, Respiratory Care Ward;**  ***the P-value <0.05 was considered statistically significant.** | | | | | | | | | | | | |
