# Supplementary material for: Stress on caregivers providing prolonged mechanical ventilation patient care in different facilities: A cross-sectional study
Source: PLoS One. 2022 May 25;17(5):e0268884. doi: 10.1371/journal.pone.0268884 (PMC9132287; doi:10.1371/journal.pone.0268884)
Supplement: Attachment — Submitted filename: renamed_90d68.docx [file pone.0268884.s005.docx]

**Response to Reviewers’ Comments**

**PLOS ONE**

**Ref. No. PONE-D-21-24193**

**Title: Stress on caregivers providing prolonged mechanical ventilation patient care in different facilities: A cross-sectional study**

**Dear Editor and reviewers:**

**We appreciated your helpful comments to improve the presentation of the paper. We have responded to all advice point by point and revised the manuscript. It is our sincere hope that this revision will enhance readability with strengthened discussions to satisfy the requirements of this prestigious journal.**

1. **Please include additional information regarding the survey or questionnaire used in the study and ensure that you have provided sufficient details that others could replicate the analyses. For instance, if you developed a questionnaire as part of this study and it is not under a copyright more restrictive than CC-BY, please include a copy, in both the original language and English, as Supporting Information.**

**Response: Thanks for your comments.**

**We provided the questionnaire in both Chinese and English (Attachment 1) and all of the questions are listed in table 3 on the main document (see page 9-11 on table 3).**

2. **Furthermore, please provide additional information regarding how participants were recruited for the study, and please ensure that you have provided sufficient detail to allow your work to be replicated.**

**Response: Thanks for your comments.**

**The researcher invited medical staff, case managers, or respiratory therapists to recruit those who fit the criteria of the subjects to accept the interview and explained to the respondents according to the interview instructions (Attachment 2). If the interviewee did not agree to accept the questionnaire survey, they could withdraw their consent from this research without any reason, with no negative consequences.**

3. **Moreover, in the Methods section, please provide a** **justification for the sample size used in your study, including any relevant power calculations (if applicable).**

**Response: Thanks for your queries.**

**Our justification for the sample size is as follows, and was edited in methods section.**

**This was a cross-sectional observational study. According to Mohamad Adam Bujang et al.'s recommendation for observational large-sample studies using logistic regression, the minimum sample size needs to be at least 500 [25]. A total of 601 valid samples were collected in this study, so there were sufficient samples (See line 103-107 on page 5).**

**We cited the reference 25 as follows:**

***Bujang MA, Sa'at N, Sidik T, Joo LC. Sample Size Guidelines for Logistic Regression from Observational Studies with Large Population: Emphasis on the Accuracy Between Statistics and Parameters Based on Real Life Clinical Data. The Malaysian journal of medical sciences: MJMS. 2018;25(4):122-30. Epub 2019/03/28. doi: 10.21315/mjms2018.25.4.12. PubMed PMID: 30914854; PubMed Central PMCID: PMCPMC6422534.***

4. **Finally, please include in the Methods section (or in Supplementary Information files) of the participating hospitals/institutions.**

**Response: Thanks for your suggestion.**

**We have uploaded the list of participating hospitals/institutions in supplementary information file (the Attachment 3) and provides a list of 64 hospital/institution names.**

5. **Please provide additional details regarding participant consent. In the ethics statement in the Methods and online submission information, please ensure that you have specified whether consent was written or verbal/oral. If consent was verbal/oral, please specify: 1) whether the ethics committee approved the verbal/oral consent procedure, 2) why written consent could not be obtained, and 3) how verbal/oral consent was recorded. If your study included minors, please state whether you obtained consent from parents or guardians in these cases. If the need for consent was waived by the ethics committee, please include this information.**

**Response: Thanks for your queries.**

**Because patients are mostly unconscious and interviewers are required to assist in collecting patients’ clinical information, the research invites medical staff, case managers, or respiratory therapists to recruit those who understand the situation of the patients to do the interview, and explains to the main caregiver of the family according to the interview instructions (see the Attachment 2).**

6. **We note that the grant information you provided in the ‘Funding Information’ and ‘Financial Disclosure’ sections do not match. When you resubmit, please ensure that you provide the correct grant numbers for the awards you received for your study in the ‘Funding Information’ section.**

**Response: Thanks for your suggestion.**

**This study was supported by grants (grant numbers DOH102-NH-9009; DMR-109-015) from the National Health Insurance Administration and China Medical University Hospital, Taiwan. The funders had no role in study design, data collection and analysis, decision to publish, or preparation of the manuscript (See line 284-288 on page 19).**

7. **Please include your tables as part of your main manuscript and remove the individual files. Please note that supplementary tables (should remain/ be uploaded) as separate "supporting information" files**

**Response: Thank you for your comments.**

**We have inserted tables in the manuscript.**

**Reviewer #1**

1. **The article is interesting one because it has looked issues from different aspects rather than medical only. It would be better if you could find out socioeconomic conditions and occupation of caregivers and underlying diseases of the patients impact on stress level and family relationship.**

**Response: We used family income and education level to represent the socioeconomic conditions of caregivers, and compared the family income and education level of the caregivers of PMV patients at four stages with the psychological pressure and family relationship with** **Spearman's rank correlation. In addition, we used the** **Chi-square test to analyze the patient's underlying disease, psychological pressure of the caregiver, and family relationships. The results showed that there was no significant difference in the family income of caregivers between psychological stress and family relationships, but it was found that the higher the education level, the lower the adverse effect on the relationship between family members, and there was a statistically significant difference (p<0.05) (Table 4). There was no significant correlation between the underlying disease of PMV patients and the psychological stress of caregivers and family relationships (Tables 5). We have revised the text in the manuscript (See table 4-5 on page 14-15.)**

2. **Page 11, last paragraph, I think it's home-based PMV rather than PMV alone.**

**Response: Thank you for your comments.**

**I really want to express home-base, and modified it as follows:**

**“The results show that caregivers of ICU and home-based PMV patients have higher stress levels than those of RCC and RCW patients.”**

**Reviewer #2:**

1. **missing tables can not make the review**

**Response: Thank you for your comments.**

**We have inserted tables in the manuscript.**

2. **Many language errors**

**Response: Thank you for your comments.**

**We send the revised file back to the translation agency for revision in English (Attachment 4).**

3. **Many abbreviation don’t correspond correctly to their phrases Like “RCW” and “RHC”**

**Response: Thank you for your comments.**

**We have revised the abbreviations of RCW and RHC in the text to make it the same as the full text. RCW, respiratory care ward; RHC, respiratory home care**

4. **“The main purpose of this study is to provide a solution for the shortage of ICU beds” That is a confusing sentence as this isn’t the aim of the study!!**

**Response: Thank you for your comments.**

**We have deleted this sentence from the introduction.**
