# Supplementary material for: Stress on caregivers providing prolonged mechanical ventilation patient care in different facilities: A cross-sectional study
Source: PLoS One. 2022 May 25;17(5):e0268884. doi: 10.1371/journal.pone.0268884 (PMC9132287; doi:10.1371/journal.pone.0268884)
Supplement: Attachment — Submitted filename: Response to Reviewers.docx [file pone.0268884.s006.docx]

**Response to reviewers’ comments**

**Reviewer #1:**

1. Abstract: Page 2, line 42-45, the sentence lacks subject

Response: Thanks for your queries.

We found no statistically significant difference at p=0.05, so we deleted this sentence in the abstract section.

2. Abstract: Page 3, line 58, caregivers home or in-home caregivers?

Response: Thanks for your comments.

Since what we are visiting is to take care of the family members of PMV patients at home, this should be in-home caregiver. We revise the sentence as “Thus, the government should provide more support systems for in-home caregivers to relieve their life stress.” (Page 3, line 50-51 in revised vision)

3. Introduction: Line 70, Studies…… This sentence is not complete.

Response: Thanks for your comments.

We are grateful for this suggestion and have completed sentences to clarify this point as follows: “Studies show that as patients who stay at home are more familiar with the environment, they feel more comfortable, and experience is better than the in-house care patients. [14].” (Page 5, line 76-78 in revised vision)

**Reviewer #2:**

1. “Home-based interviewees reported that taking care of the patient of their family worsens the relationship between family members than the intensive care unit group interviewees” What do you mean exactly by this result in the abstract, are you pointing to family member giving care to chronic ventilated patients and the consequences or the respiratory caregiver and the relation to the family?!

Response: Thanks for your queries.

We have revised the result of abstract as follows: “As the results of the life impact presented in the family domain that RHC interviewees (OR=2.54) feel it worsens family relationships than the ICU group; however, there is less psychological stress in RCC (OR=0.54) and RCW (OR=0.16) than ICU group. In the social domain that RHC interviewees feel reducing friend/family interactivity (OR=2.18) and community/religious activities (OR=2.06) than ICU group; however, RCW interviewees fell less effect with leisure time (OR=0.37) and work time (OR=0.41) than ICU group. Furthermore, the economic domain of reducing family income because caregivers cannot work is less influenced in RCW interviewees (OR=0.43) than in ICU groups.” (Page 2, line 39-48 in revise vision)

2. In the abstract after that sentence you switch to stress as consequence of this relation to family You conclude in the abstract that “primary family caregivers of patients with prolonged mechanical ventilation had the highest level of stress among in-home caregivers ”I assume those are respiratory therapist handling patients at home as you r inclusion criteria is of healthcare professional only not family members taking care of relative on home ventilator, but in the results you mention nothing about their stress level, only you mention about their relation with family members
   Response: Thanks for your queries.

We have revised the conclusion in abstract as follows: “Stress levels of primary family caregivers of patients with prolonged mechanical ventilation had the highest stress level among in-home caregivers and less in the RCW group. Thus, the government should provide more support systems for in-home caregivers to relieve their life stress.” (Page 2-3, line 66-69 in revised vision)

3. By the end your abstract isn’t well written and the results aren’t summarized in good way for the reader to perceive the message.

Response: Thanks for your comments.

We have major revision in the abstract (Page 2-3 line27-51, in revised vision).

4. Your population isn’t clear or defined especially regarding those involved in RHC (respiratory home care)

Response: Thanks for your comments.

We have revised the introduction as follows:

“The regulations of IPP are fee for service for ICU care (within 21 d), per-diem for RCC (for up to 42 d), per capitation for RCW, and monthly payment for home ventilation service. The ICU, RCC, and RCW belong to the institution care, and 24-hours medical staff is available. The patients who use ventilators at home have nurses’ and respiratory therapists’ visits twice a month and physicians’ visits once two months.” (Page 4 line 69-73, in revised vision)

5. The objective of your study isn’t clear “We measured aspects related to their lives using a Likert scale”

Response: Thanks for your queries.

We have revised the part of questionnaire development in Materials and Methods section as follows:

“The questionnaires have three domains (family, social, and economic aspects) that were measured related to their lives using a Likert scale (from "strongly agree" to "strongly disagree") to evaluate the impact of the IPP and the stress of life on main caregivers of PMV patients at different units.” (Page 6 line 102-105, in revised vision)

6. Where’s the detailed table showing the numbers of respondents in the different subcategories based on the variables you measured, it seems based on table one that all or most of population were from RHC.

Response: Thanks for your queries.

The numbers of respondents in the different subcategories were showed in table 2. A total of 601 interviewees were analyzed, and they consisted of ICU (150), RCC (150), RCW(150), and RHC(151). (Page 9-10, table 2, line 182-185, in revised vision)

**Response to the Editor’s comments**

1. Please include captions for your Supporting Information files at the end of your manuscript, and update any in-text citations to match accordingly. Please see our Supporting Information guidelines for more information: http://journals.plos.org/plosone/s/supporting-information.

Response: Thanks for your comments.

We added our supporting information at the end of our manuscript, and please see line 290-296 on page 20. The supporting information is as follows:

S1 File: Question about the impact of taking care of prolonged mechanical ventilation patients on life (English and Chinese version)

S2 File: Interviewees Manual (English and Chinese version)

S3: 64 Interviewee's institutions information

S4: The impact on the family caregivers’ life between the ICU, RCC, RCW and RHC groups

2. We note your Data Availability statement:

"All relevant data are within the table 1-5, the raw data can’t share public due to the data contain personal information. This study was approved by the Institutional Review Board of China Medical University Hospital (IRB No. CMUH102-REC3-105), Taiwan." According to PLOS policy, when specific legal or ethical restrictions prohibit public sharing of a data set, authors must indicate how others may obtain access to the data. Authors must share the “minimal data set” for their submission. PLOS defines the minimal data set to consist of the data required to replicate all study findings reported in the article, as well as related metadata and methods. Please see this link for more details: <https://journals.plos.org/plosone/s/data-availability#loc-minimal-data-set-definition> If the minimal data set is contained within the raw data, please update your Data Availability statement with a non-author contact who can provide these data upon request. This could be a contact at the Institutional Review Board of China Medical University Hospital, Taiwan, or other such body

Response: Thanks for your comments.

We have added the supporting file (S4 file) for raw data of the figure 1-3. If you have any questions related to the minimal data set, please let us know.

**Author’s Statement**

1. We are sorry that we found that the first question of the economic domain in figure 3 was misquoted the fifth question of the social domain in Table 3. We have revised it.
