# Supplementary material for: Stress on caregivers providing prolonged mechanical ventilation patient care in different facilities: A cross-sectional study
Source: PLoS One. 2022 May 25;17(5):e0268884. doi: 10.1371/journal.pone.0268884 (PMC9132287; doi:10.1371/journal.pone.0268884)
Supplement: Attachment — Submitted filename: Response to Reviewers.docx [file pone.0268884.s007.docx]

PONE-D-21-24193R2

Stress on caregivers providing prolonged mechanical ventilation patient care in different facilities: A cross-sectional study

1. Reviewer #2: The paper is still confusing, and mixed up, still you confuse the family caregiver with the respiratory therapist in your abstract and manuscript, to the degree I am not sure the result belong to whom, please revisit your conclusion in the abstract, this is after 2 runs of revision. I cant get the theme from the paper and conclude main outcome or idea.

**Ans.:** We feel very sorry for the confusion. We have revised and marked with red color for revision in the manuscript. Thank you for reminding and providing the opportunity to revise.

**Abstract (Revised)**

**Purpose:** Taiwan has implemented an integrated prospective payment program (IPP) for prolonged mechanical ventilation (PMV) patients that consists of four stages of care: intensive care unit (ICU), respiratory care center (RCC), respiratory care ward (RCW), and respiratory home care (RHC). We aimed to investigate the life impact on family caregivers of PMV patients opting for a payment program and compared different care units. **Method:** A total of 610 questionnaires were recalled. Statistical analyses were conducted by using the chi-square test and multivariate logistic regression model. **Results:** The results indicated no associations between caregivers’ stress levels and opting for a payment program. Participants in the non-IPP group spent less time with friends and family owing to caregiver responsibilities. The results of the family domain show that the RHC group (OR=2.54) had worsened family relationships compared with the ICU group; however, there was less psychological stress in the RCC (OR=0.54) and RCW (OR=0.16) groups than in the ICU group. In the social domain, RHC interviewees experienced reduced friend and family interactivity (OR=2.18) and community or religious activities (OR=2.06) than the ICU group. The RCW group felt that leisure and work time had less effect (OR=0.37 and 0.41) than the ICU group. Furthermore, RCW interviewees (OR=0.43) were less influenced by the reduced family income than the ICU group in the economic domain. **Conclusions:** RHC family caregivers had the highest level of stress, whereas family caregivers in the RCW group had the lowest level of stress.
