# Supplementary material for: Stress on caregivers providing prolonged mechanical ventilation patient care in different facilities: A cross-sectional study
Source: PLoS One. 2022 May 25;17(5):e0268884. doi: 10.1371/journal.pone.0268884 (PMC9132287; doi:10.1371/journal.pone.0268884)
Supplement: Attachment — Submitted filename: Response to Reviewers comments.docx [file pone.0268884.s008.docx]

**Response to reviewers’ comments:**

PONE-D-21-24193R3

Stress on caregivers providing prolonged mechanical ventilation patient care in different facilities: A cross-sectional study

Reviewer #1:

The article will be very useful for deciding site of care for patients who need long term ventilatory support. The findings may serve as issues for discussion.

Ans.: Thanks for your comments.

Reviewer #3:

1. Line 66:“The ICU, RCC, and RCW is belonging the institution” –- suggest English consultation “
   Ans.: Thanks for your comments.
   We are grateful for this suggestion and try to clarify this sentence, which was as follows “The ICU, RCC, and RCW belong to the institutions,” (Page 3, line 67 in revised vision)
2. Line 97:“The questionnaires have three domains (…) was measured aspects related to their lives by a Likert scale(…) to compared with the Impact of the IPP and different unit of main caregiver of PMV patients on the stress of life. ” –- suggest English consultation

Ans.: Thanks for your comments.
We are grateful for this suggestion and try to clarify this sentence, which was as follows “The questionnaires have three domains (family, social, and economic). They measured aspects related to their lives by a Likert scale (from strongly agree to strongly disagree) to compare the impact of the IPP and different units of primary caregivers of PMV patients on the stress of life.” (Page 5, line 97-100 in revised vision)

3. Line 113:“the acute ICU stage was the time or less than 7 days in RCC after transfer from ICU;”→-“the acute ICU stage was the time in ICU and/or less than 7 days in RCC after transfer from ICU;”---???

Ans.: Thanks for your comments.

We are grateful for this suggestion and try to clarify this sentence, which was as follows “the acute ICU stage was the time in ICU and/or less than 7 days in RCC after transfer from ICU” (Page 5, line 113-114 in revised vision).

4. Line 143:“The monthly expense for the IPP group was significantly lower than that for the non-IPP group (USD 745 vs. USD 912 respectively) (Table 1).” --- The definition of “monthly expense” is not clear. Is it the expense paid by the family or by the Taiwan National Health Insurance Program?

Ans.: Thanks for your comments.

We are grateful for this suggestion and try to clarify this sentence, which was as follows “The monthly expense paid by the family for the IPP group was significantly lower than that for the non-IPP group (USD 745 vs. USD 912, respectively) (Table 1).” (Page 7, line 145 in revised vision).

5. Line 144:“the non-IPP group” --- The recruitment of “non-IPP group” was not described in the methodology.

Ans.: Thanks for your comments.

We are grateful for this suggestion and have added sentences to clarify this

point, which were as follows “The participants were divided into two groups (IPP and none-IPP group) according to whether they had joined IPP.” (Page 5, line 117-118 in revised vision).

6. Line 148 Table 1 --- The last line of 1st column“Average monthly expense(SD) (USD) “→“Average monthly expense (USD) Mean (SD)
   Ans.: Thanks for your comments. We have changed the description. (Page 8, line 151 the last line of 1st column in revised vision).
7. Line 193, 208, 216 “Odds of agree in the life impact” →“Odds of agreement in the life impact”
   Ans.: Thanks for your suggestion. We have changed the description. (Page 15, line 196; Page 16, line 211; Page 16, line 219 in revised vision).
